# Barriers to providing healthcare to children living with cerebral palsy in Ghana: A qualitative study of healthcare provider perspectives

**DOI:** 10.1371/journal.pgph.0001331

**Published:** 2022-12-09

**Authors:** Habibat A. Oguntade, Thamanna Nishath, Prince G. Owusu, Christina Papadimitriou, Kwame S. Sakyi

**Affiliations:** 1 Center for Learning and Childhood Development-Ghana, Accra, Ghana; 2 Division of Epidemiology and Community Health, School of Public Health, Minneapolis, University of Minnesota, Minneapolis, Minnesota, United States of America; 3 Department of International Health, Johns Hopkins Bloomberg School of Public Health, Baltimore, Maryland, United States of America; 4 University of Washington School of Medicine, Seattle, Washington, United States of America; 5 Department of Human Development and Family Studies, Michigan State University, East Lansing, Michigan, United States of America; 6 Department of Interdisciplinary Health Sciences and Sociology, School of Health Sciences, Oakland University, Rochester, Michigan, United States of America; 7 Department of Public and Environmental Wellness, School of Health Sciences, Oakland University, Rochester, Michigan, United States of America; MCRI, University of Melbourne, Royal Children's Hospital Melbourne, AUSTRALIA

## Abstract

Children with neurodevelopmental disabilities in low- and middle-income countries (LMICs) experience profound health and social inequities. While challenges faced by children living with disabilities and their caregivers have been widely documented, little is known about barriers faced by healthcare providers (HCPs) who serve these children. This study seeks to understand the barriers to testing, diagnosing, referral, and treatment of children living with cerebral palsy (CLWCP) from the perspectives of HCPs in Ghana. This qualitative study was conducted in the Greater Accra region of Ghana. A snowball sampling strategy was used to recruit HCPs from major hospitals, education centers, and health facilities. Data were collected through 11 semi-structured in-depth interviews (IDIs) with HCPs. Using an adapted version of the Sweat & Denison socio-ecological framework (SDSF), barriers to providing healthcare to CLWCPs were organized into superstructural, structural, environmental, relational, individual, and technological levels. We found that barriers to providing healthcare to CLWCPs exist at all levels of the adapted framework. The most salient barriers were identified at the superstructural, structural, and environmental levels. All HCPs expressed frustration with Ghana’s health insurance policies and inadequacies of the health systems infrastructures, such as patient assessment rooms, health information systems, and pharmaceutical products for CP care. HCPs also reported that disability-related stigma often discourages providers in training from specializing in the area of developmental disabilities. HCPs emphasized critical challenges related to local perceptions of disability, gender norms and ideologies, and health system policies and infrastructure. Findings highlight the importance of identifying multi-level factors that can influence testing, diagnosing, referral, treatment, and provision of care for CLWCPs in Ghana. Addressing identified challenges from each level of influence may improve CLWCP’s experiences throughout the care continuum.

## Introduction

Worldwide, an estimated 1 billion (15%) people live with some form of disability [1]. Specifically, the number of children between the ages of 0 and 14 years experiencing moderate or severe disability is about 93 million (5.1%), and those experiencing severe disabilities is 13 million (0.7%) [1]. Cerebral palsy (CP) is the most common physical disability among children, with an estimated prevalence of 2–2.5 per 1,000 live births, which is suggested to be higher in African countries due to underreporting [2–6]. CP is defined as “a group of permanent disorders of the development of movement and posture causing activity limitations that are attributed to non‐progressive disturbances that occurred in the developing fetal or infant brain,” and it presents on a spectrum of manifestations [7, 8]. However, this definition only reflects the medical model of disability, which views impairments as the main cause of disability and primarily focuses on underlying biological causes [9]. Alternatively, the social model of disability posits that the notion of disability is constructed by society and offers a view that looks beyond an individual’s impairment. It provides a nuanced approach to understanding experiences of inequality among people with impairments by focusing on how the social environment deprives them of equal opportunities to participate in society [10]. Therefore, the social environment, not biological factors, is what disables an individual.

Studies show that children living with CP (CLWCP) in low- and middle-income countries experience a significantly poorer health-related quality of life and increased mortality rate, high burden of disease with preventable risk factors, and the experience of stigmatization and discrimination [11–16]. In Ghana, for example, one study found that the standard mortality rate of children with CP is six times that of the average Ghanaian child without a disability and 15 times that of the average child without a disability in a low-and-middle-income country [17]. According to another study, along with most CLWCPs having severe forms of the disease, Benin also has a high prevalence (17%) of the rare post-neonatal type of CP and low school attendance among its CP population [18]. Uganda similarly has high rates (25%) of postnatal cerebral palsy, largely attributed to infectious causes, which highlights the disproportionate disease burden and factors influencing low resource settings [19]. A study from Ghana found that many, if not all, primary caregivers of CLWCPs faced discrimination and neglect from families and their communities, incurred financial hardships, and had difficulty accessing suitable educational and medical facilities for their CLWCP [20]. These poor outcomes point to persistent structural barriers, inadequate healthcare and social services, and limited access to and use of evidence-based interventions, such as assistive technology [11–13]. To bridge the inequities, it is critical to examine challenges in the testing, diagnosing, referral, and treatment of CLWCPs in LMICs to inform policy and interventions.

### The Ghanaian healthcare system and care for children with cerebral palsy

The Ghanaian National Health Insurance Scheme (NHIS) was established in 2003 to provide access to basic health services. It has contributed to improved health services utilization and health outcomes as it moves towards achieving universal health coverage [21, 22]. The NHIS covers primary healthcare (PHC) services, with benefits packages covering almost 95% of Ghana’s disease burden and various outpatient and inpatient services, from maternity care to oral health services [23, 24]. The NHIS is free for children below 18 years of age with a beneficiary parent, adults over 70 years of age, individuals with mental health conditions, pregnant individuals, and those without a source of income, but requires adults between 18–69 years to pay a yearly premium [23, 24].

Unlike individuals with higher socioeconomic status, many people with low income remain uninsured due to the inability to afford the premium required by the NHIS [21–23, 25, 26]. Ghanaians with lower socioeconomic status are less likely to be covered by the NHIS and, thus, more likely to experience financial difficulties due to out-of-pocket (OOP) payments [24, 27–30]. Some initiatives have been developed to offset the high cost of health services for low-income individuals in Ghana [24, 27–30]. An example is the Livelihood Empowerment Against Poverty (LEAP), a cash and health insurance transfer program for poor households to combat short-term poverty and contribute to long-term human capital development [31–33]. Despite such efforts, the number of families in need far surpasses the availability of services and access to care, while appropriate health services remain inaccessible and unaffordable [17]. Ghana’s medical system is heavily centered around its two largest cities, Accra and Kumasi, with almost 3 million inhabitants accessing the majority of physicians, up-to-date medical resources, and varied health services. This is not the case in many rural areas, negatively impacting CLWCPs [15, 32].

One systematic review identified 15 evidenced-based, high quality standards of care interventions for CP, ranging from medications to movement training [34]. In Ghana, the Princess Marie Louise Children’s Hospital has a standard of care for pediatric CP that includes functional training at the physiotherapy clinic three times a week, a collaboration between pediatricians and physiotherapists for medication dosage, and visits to the nutrition clinic for adequate dietary intake and education for caregivers. In tandem, many caregivers of CLWCPs participate in community support groups that serve as emotional support systems and provide advice on day-to-day care management [31, 35]. Caregivers also rely on alternative medicine, such as herbs or spiritual healing, due to the desire for a permanent cure for CP, symptoms that were not sufficiently addressed by the medical system, perceptions about disability, cost, family referral, and poor provider relationship [36]. Despite the availability of a few services, studies show that finding providers who can offer the specific interventions that CLWCPs need can be challenging for caregivers in low-resource settings [37, 38].

### Gaps in literature

Most of the studies on CP have been conducted in high-income countries with limited evidence from low- and middle-income countries (LMICs) [2, 5, 38–40]. A systematic review of 49 studies on the prevalence of CP between 1996 and 2013 included few studies from LMICs, with a majority originating from North America or Europe [41]. Another systematic review on quality of life among CLWCPs in LMICs found only 16 studies from 8 LMICs [13, 41]. Notwithstanding these reviews, there is a general scarcity of information about CP from LMICs and a few studies exist on general neurological diseases that include CP peripherally [42, 43]. A cross-sectional survey examining the prevalence of disabilities among children in the Central Region of Ghana concluded that 1.8% of children under fifteen years of age had disabilities, which is equal to a prevalence of 14.4 per 1,000 for children ages 1–5 years, 16.6 per 1,000 for children ages 6–9 years, and 3.7 per 1,000 for children ages 10–15 years [44]. Outside of this study, there is insufficient data on the prevalence and treatment of childhood developmental disabilities like CP in Ghana.

Besides the geographic limitation of the existing studies, most studies of CP in Africa have mainly focused on mobility-related issues and experiences of caregivers [31, 45–48], and relied mainly on a quantitative methodology [45]. Together, these studies suggest that caregivers of CLWCPs often experience multi-level challenges during, before, and after CP diagnosis. For example, many caregivers feel negative emotions and pressure to keep their children out of the public eye due to social misconceptions, stigma, and discrimination in their communities [15, 49]. This often leads to delayed care-seeking and reduced access and utilization of essential health and social services [47, 50]. A study of parents with CLWCPs found that over 60% of these caregivers were nervous or stressed and had a negative correlation between psychological distress and good social support, indicating an unmet need for parental support [2, 49]. Additionally, caregivers also experience health systems challenges, such as a lack of access to specialist clinics and negative encounters with health professionals [48].

Care management for CLWCPs goes beyond the household and should be assessed from multiple perspectives. Nevertheless, a few studies have examined the challenges specialist providers of CP face in sub-Saharan Africa, and the countries within. CLWCPs often need highly specialized care that requires a multidisciplinary group of providers and caregivers, such as pediatricians, general practitioners, orthopedic surgeons, nutritionists, speech and language therapists, occupational therapists, and physiotherapists [51, 52]. The existing literature suggests that CLWCPs may face challenges at many levels, including the home, community, clinical, and policy level. As a result, an investigation of the experiences of specialist providers should take a multi-level approach.

### Study aims

The aim of this study was to examine the barriers to testing, diagnosing, referral, and treatment of CP from the perspective of specialist healthcare providers in Ghana.

## Methods

### Ethics statement

The study was approved by the Ghana Health Service Ethical Review Committee [GHS-ERC 08/08/16]. All potential participants were informed about the study objectives and data collection activities. They were also given an opportunity to ask questions and obtain clarification on study procedures to make an informed decision about participating. Only individuals that provided oral and written consent were invited to participate in the study.

### Study setting and participant selection

The study was conducted in partnership with the Center of Learning and Childhood Development, Ghana (CLCD). CLCD is a local non-profit organization that aims to improve children’s survival and development through research, advocacy and practice, and capacity building. CLCD has been instrumental in designing childhood development interventions within the Greater Accra region. The Greater Accra region is the most populated region in Ghana, with over 4 million people and has the greatest number of specialist healthcare providers in the country. We defined our target population as healthcare providers (HCPs) who provide specialized care for CLWCPs in the Greater Accra region of Ghana. Participants were eligible for the study if their work involved any form of examining, diagnosing, treating, or providing services to CLWCPs. A snowball sampling strategy was used to recruit HCPs from major hospitals, education centers, and health facilities in the Greater Accra region of Ghana. This sampling method was selected to identify information-rich participants considering the limited number of HCPs who care for CLWCPs in Ghana. Throughout this paper, we will refer to study participants as HCPs.

### Inclusivity in global research

Additional information regarding the ethical, cultural, and scientific considerations specific to inclusivity in global research is included in the S1 Text.

### Study design

We developed an in-depth interview (IDI) guide to assess barriers related to different categories of healthcare management for CLWCPs. The IDI guide was designed to be administered through one-on-one interviews by one interviewer. It was pilot tested with developmental disability specialists who were not part of the study participants. Questions in the IDI guide focused on barriers within the categories of testing, diagnosing, referral, and treatment of CP. Some examples of topics discussed were types of tools and instruments used in diagnosing CP, affordability and accessibility of treatments, and continuity of care. Qualitative data were collected through IDIs with HCPs in the Greater Accra region of Ghana between June and August 2017. The reporting of our study methodology follows the consolidated criteria for reporting qualitative research (S1 Checklist) [53].

### Data collection

The 1^st^ author, HO, (B.A, B.S. MSPH) who identifies as a female, conducted the semi-structured IDIs with participants. At the time of the study, HO was a second-year Master of Public Health student with extensive experience in conducting qualitative interviews with vulnerable populations. KS, (MSPH, PhD) was a trained social behavioral scientist. PO, (MEd, BSc), who supervised data collection, was the co-founder and the director of operations at the CLCD. PO had over seven years of experience working with families of children with CP, schools and child development professionals in Ghana. KS and PO have worked on multiple projects involving caregivers and health professionals who provide healthcare to CLWCPs in Ghana. KS and PO trained HO in conducting interviews using strategies appropriate for the study population. At the beginning of each interview, HO established rapport with the participant by discussing her academic background and how her research interests were shaped by her experiences as a Nigerian. Then, she briefly described the study and obtained informed consent from all HCPs. The interviews were conducted in private offices or locations the HCPs chose, and only the interviewer and participants were present for all interviews. After each interview, the research team (HO, KS, and PO) had a debriefing session where they discussed the data collection process and emergent themes. KS and PO also listened to some of the interview audio recordings to identify strategies to improve quality of interviews. The number of IDIs conducted was determined by the limited number of health professionals who provide healthcare to CLWCPs. Furthermore, after interviewing eleven participants, the team decided to end data collection as saturation was reached as the information gathered from participants became repetitive. IDIs were conducted in English and lasted for approximately 60 minutes per interview. IDIs were audio-recorded (with permission from participants) and transcribed verbatim. Handwritten notes were also taken during the interviews.

### Data analysis

The analysis process occurred in four main steps. The first step began with a close reading of all interview transcripts to gain familiarity with the data. Transcripts were imported to ATLAS.ti Mac (Version 8.4.5) to organize and code data. In step two, HO coded two interview transcripts using a thematic content analysis approach [54]. Then, a deductive approach [55] using pre-determined categories from the in-depth interview guide was used to generate initial codes (such as wait-times, out-of-pocket costs, cultural perceptions etc.) that represented notable features across transcripts. Several meetings were held with the study team to discuss the initial codes and make modifications where needed. Using the revised codes, HO developed the initial codebook that included a codename, a brief definition of the code, and when to use the code. The codebook was then reviewed and revised by the study team before it was applied to the remaining interview transcripts.

The third step involved reviewing the coded barriers and grouping the barriers into categories (themes) based on their similarities and differences. The themes were further coded to delineate the relationship between themes. Memos and discussions with the research team were also used to refine the linkages between specific themes. The emergent results showed that barriers to providing healthcare to CLWCPs existed on multiple levels. Therefore, the identified barriers were organized around the Sweat and Denison socio-ecological framework (SDSF) [56]. This framework was chosen because the descriptions of the different levels closely reflected the themes identified in this setting. In addition, the SDSF’s major strength is that it creates an avenue to engage multiple stakeholders (e.g., policymakers, health professionals, community members etc.) to identify mechanisms of change, an approach needed to address the multi-faceted barriers identified in this study. Although the SDSF was originally designed for HIV prevention interventions, it provides a unique opportunity to examine the relationship between these levels of influence and how they relate to several health problems. The framework has four levels of influence: superstructural, structural, environmental, and individual [56]. Despite its strengths, not all the emerging themes fit into the existing SDSF levels. Thus, the model was adapted by adding relational and technological levels (see Table 1).

**Table 1:** Multi-level approach to identifying barriers to testing, diagnosing, referral, and treatment of CLWCPs in Ghana–A social ecological framework adapted from Sweat and Denison.

Level	Definition	Examples	Supporting Quotes
Superstructural	Macrosocial and macropolitical arrangements, resources and power differences that could result in unequal advantages for people living with disabilities	Gender imbalances, income levels	*“What I have found is that a lot of the people that we see here [clinic]- a lot of people who bring in their kids*, *a lot are the mothers*. *So*, *the men are often not really involved in the intervention*. *I can count the number of men we see in clinic*, *really*. *So*, *[according to the men] ‘it is the women who have done this*, *it is you [the woman] who have carried the child for nine months*, *so this must be coming from you and not from me*,*’ you know*? *And so*, *‘you must be responsible for this*.*’ There are those [men] who even*, *because of that*, *leave the marriage*. *And so*, *women become single parents*, *with the burden of having to afford a child with a disability*.*” (IDI 9)*
Structural	Laws, policies, and resources that impacts how medical and social systems operate	Health systems infrastructure, insurance policies, social services	*“The second challenge is with records*, *computerization of records*. *So*, *you came*, *and you want data*, *everyone likes that*, *but we have not invested in collecting accurate data*. *We write all these information in folders*. *Now*, *when you go to the clinic*, *I have like 4000 folders for cerebral palsy or epilepsy*, *that is a lot of data is it not*? *If you want to know about epilepsy or cerebral palsy in Ghana*. *But you see it is all handwritten*. *It is not computerized*. *So*, *when you ask me*, *how many children with new onset epilepsy have you seen this year*, *oh I just have to try to remember what I have seen*. *But what have the others seen*? *Data needs to go into computer soon after a clinic so that we can come up with prints*, *we can research and publish more papers from our side of the world because that is what is lacking*. *If you go most times for the literature search*, *most times you will notice that there is a scarcity of research data from West Africa or Africa*, *though we have very good clinical cases and you know I always say this place is like a museum*, *any case you want to see*, *you will see it*, *alright*, *but are we known in the international scene*? *No*.*” (IDI 6)*
Environmental	Individual living conditions, shared expectations, and rules of how people behave and interact with their physical or social environment	Community perceptions, community support, social norms, stigma, discrimination	*“In the Ghanaian culture a child with cerebral palsy is perceived to be representing spirits which has resulted in the mother giving birth to that kind of child*. *That the child came from a spirit*, *an evil spirit and that’s where it has manifested in that kind of child*. *So anytime a child is seen to have cerebral palsy*, *they think their child is from the spirits*. *I grew up in a very remote area where there was this child with cerebral palsy…*.. *No one played with the child*, *but I had this love for the child*, *and I used to sit with her and play with her*. *I recently got to know that the child had been dumped in the stream*. *The child had been left in the stream for dead*. *So that was very*, *very bad…*. *I went back and I asked and was told the child had been sent off to the spirits*.*” (IDI 4)*
Relational	Interactions between caregivers and healthcare providers that influence care seeking, care management and continuity of care	Communication, expectations, trust	*“Well*, *it [local perceptions of disability] is a big challenge because*, *like I said*, *most of them [CLWCPs] come very late to us because they don’t know that these problems are psychiatric problems or health-related problems*. *So*, *they go to several other places before they get to us*. *Because their illness perception*, *some think it’s spiritual*, *some think it’s a curse*. *All kinds of things*. *So*, *then they come to us late*, *and once the child comes late*, *it limits the number of things that you are able to do for the child*. *So*, *that’s a big issue; there’s always this*.*”* (IDI 7)
Individual	How the different levels are experienced, internalized and acted upon by individuals	Attitudes, knowledge, self-efficacy	*“There are very few doctors who are very comfortable in the area of neurodevelopment*, *especially when it comes to children*. *I think in the area of neurodevelopment*, *the specialist needs to a have certain level of training*.*” (IDI 1)*
Technological	Technological resources that could improve access to and quality of health services	Diagnostics tools, medical devices, telecommunication devices	*“And there are some cases that you see and know that the person will benefit from cochlear implants*. *But it is not done here*, *and the cost involved currently is an estimate of 45*,*000 [45*,*000 GHS is approximately 6*,*000 USD] from South Africa for one implant*. *Your plane fare*, *accommodation*, *you may need to go for review every 3 months*, *for some time*. *So*, *you [the specialist] just have to let the person [CLWCP]go to a special school*, *get to learn the sign language*, *but you know that this one [unique CP case]*, *with a cochlear implant will do well but you cannot do much about it*.*” (*IDI 8)

Finally, the team reviewed the results to ensure consistency with the study aims and analysis objectives as the last step. The results of the study were fat-checked in two ways. The first was through a community event where the results were shared with a CP-related mother support group. The findings resonated with them and they only shared personal stories to highlight and affirm what the HCPs said. The second was through a documentary [57] the research team produced to shift perceptions of children with CP, which involved several deliberations with caregivers and people in the disability community. The documentary provided an opportunity to conduct member-checking with community members by discussing preliminary findings of the study.

## Results

### Overview

A total of 11 IDIs were conducted with HCPs. They included an audiologist, child psychiatrist, general pediatrician, neurodevelopmental pediatrician, neurologist, occupational therapist, and speech and language therapist. The barriers to testing, diagnosing, referral, and treatment of CLWCPs in Ghana are outlined in Table 1. Emergent themes from the interviews with illustrative quotes are organized according to superstructural, structural, environmental, relational, individual, and technological levels. Although HCPs mentioned barriers that exist in all six levels of the adapted framework, the superstructural, structural, and environmental levels were the most emphasized in their interviews. Each level presents a unique barrier that impacts testing, diagnosis, referral, or treatment of CP in Ghana. However, it is important to note the interconnections between these levels, as factors interact within them to influence the barriers mentioned across the framework.

### Superstructural barriers

The superstructural level consists of “social and political arrangements, resources, and power differences that result in unequal advantages” that may lead to barriers to providing healthcare to CLWCPs [56]. Several HCPs reported that gender “imbalances” (which they described as the unequal power representation of men and women in Ghanaian society) influence care management for CLWCPs. They mentioned that women are often blamed for having the hereditary gene that causes a child’s disability. Often, these women are left alone to care for the child. HCPs also emphasized that the situation where mothers are blamed and left alone to care for CLWCPs is common among households with lower educational attainment and socioeconomic status. A female audiologist mentioned how the combined effect of these power differences, limited education, and income level can serve as a barrier to care-seeking for CLWCPs. She emphasized that the OOP cost associated with accessing treatment options are especially common barriers among parents with low socioeconomic status, tracing it to poverty. The audiologist stated that:

“*Most of our women who come*, *you ask of the husband*, *they say he has left because he claims there is no deaf person in his family… We have a lot of women… once the child is born and they [the children] show some signs of maybe Down Syndrome or cerebral palsy*, *they [the husbands] feel the child is not “normal” they feel the child is not behaving in a certain way*. *Then they just leave*, *they abandon the mother and the child*. *So now*, *to refer such a person to come for a test [is a problem]*. *Already the financial burden is all on the single mother who may not be having any [sic] better job*. *When they come*, *because we have a lot of referrals coming from outside*, *we decide to just go and see what we can do*. *Some come*, *and after they [have] paid for the tests*, *they do not have transportation to go back*. *We have to give them something to get transportation*. *(IDI 8)*

*“The issue is if I have seen you [patient] today*, *I will not see you next week to continue with it [care]*. *If you don’t have money*, *you are not going to show up next week*. *So*, *there is that issue with continuity of services*, *you know*, *a continuance of intervention*. *And you [the caregiver] would be home thinking about how to get the money*, *that maybe*, *if you had to do specific things [clinical interventions] with the child*, *they may not do it because they are thinking of how to get the money*, *how to get here [clinic] to do these things*. *And so*, *we probably may not see progress*, *or maybe minimal; whereas if the money was there and they were able to come every day*, *you would probably see a lot of changes or improvements*. *…depending on the child*, *it does play a major role*, *but everything really is about the money*, *is it not*? *Yeah*.*”* (IDI 9)

### Structural barriers

The structural level includes laws, policies, health systems, and infrastructure that could impact providing healthcare to a CLWCP. HCPs reported various health system barriers that impact testing, diagnosing, referral, and treatment of CP. This is one of the two levels where participants had the most barriers to discuss. The barriers discussed broadly centered on three interrelated issues that acted in synergy to affect the delivery of services: (1) limited health insurance policy, (2) severely inadequate human resources, and [58] (3) limited health infrastructure tailored to CLWCP.

All participants were frustrated with Ghana’s health insurance policies and inadequacies of the health systems infrastructures like patient assessment rooms, health information systems, and pharmaceutical products for CP care. Several HCPs mentioned that many of their services to CLWCPs, such as diagnostic tests and medications, are not covered by the Ghana NHIS. As a result, families of CLWCPs who are unable to afford the payment are often lost to follow-up or discontinue care. A female speech and language therapist said:

“*First of all*, *I think the biggest challenge for me is about the cost*. *So*, *if the governments can*… *If our services can just be catered for by the health insurance*, *I think it will be a big thing for our clients*, *as well as ourselves*.*” (IDI 9)*

Related to human resources, all the HCPs mentioned heavy workload, limited number of specialists, and a lack of multidisciplinary teams to collaborate pose challenges to testing and diagnosing developmental disabilities like CP. All the HCPs understood that CLWCPs required specialized care and interaction; however, due to having too many responsibilities and limited staffing, they often prioritize emergency patients, ask others to reschedule appointments, or work extremely late hours to attend to all patients. The HCPs narratives indicate that the scarcity of specialists is partly linked to the limited number of facilities that cater to CLWCPs in Ghana. Several participants described that many new graduates are unable to find a facility to practice upon graduation. Therefore, they often leave the field of developmental disability to find other jobs. A female pediatrician said:

“*The first major challenge is the load*. *It’s heavy*. *When you got a heavy load*, *you have got to work through them because you know*, *they [CLWCPs and their caregivers] have come from afar*. *In the past*, *I used to even work right up to 8pm*. *Because there are very few of us on the ground*. *VERY very few of us and at any point in time*, *we are ‘soooo’ tied down in other areas that it becomes difficult to say okay for today maybe the three of us are sitting down and running a clinic*. *Our services will be needed on the ward*, *in the emergencies and other places*. *So*, *it’s usually one-on-one consultation and not with a team of specialists*. *In this facility for instance*, *there are only three of us*. *And it’s not three of us for the neuro clinic*, *but three specialists for clinical care in the emergency*, *in the ward*, *at the outpatient department (OPD)*, *so it’s quite busy*.*” (IDI 1)*

The health systems infrastructure concerns which participants pointed out included outdated patient medical records, lack of labs and lab equipment, and lack of access to mobility devices. Most health facilities, the HCPs described, still utilize paper-based data capturing tools, which makes it extremely difficult to access and retrieve relevant and timely information on neurodevelopmental disabilities. In addition, because most facilities lack labs and lab equipment to run diagnostic tests in-house, they often have to send their patients to multiple locations to conduct tests. These errands are not always feasible for caregivers because of transportation costs and long wait times. Compounding this is the lack of access to mobility devices like wheelchairs, which makes it difficult for caregivers to transport CLWCPs to several locations, especially CLWCPs who are unable to walk. A female child psychiatrist expressed:

“*The major challenge is that the facility is not well-adapted for caring for children with special needs because we do not even have a separate room for the children*. *Of course*, *obviously seeing a child is different from seeing an adult*. *So*, *you need to have a room which is adapted to their needs*: *where they can have a play day*, *but we do not have any of those things*. *Then*, *we also do not have the full complemental services*. *So*, *most of the time*, *they [CLWCPs] have other problems aside from the behavior problems that we need to manage*, *so they may need occupational therapy for some of them have not been in school; they may need speech and language therapists; some of them may need physiotherapy services*. *And*, *to deliver good service*, *we need all the support in one place*, *so that if I need somebody to attend to deafness*, *I do not have to send a patient to another hospital*. *So that’s our—biggest problem*: *we do not have all the services in one place*, *so the child doesn’t get the full service*. *Sometimes we send them to another facility*, *and they do not even go because it can take another hour or an hour and a half to get there*. *They may have to go there on another day*, *so that is an extra cost*. *But if we had everybody [health specialists] in one place*, *then it is easier*. *So*, *yes*, *the biggest issue is the facilities*.*” (IDI 7)*

### Environmental barriers

The environmental level consists of living conditions, shared expectations, and rules of how people behave and interact with their physical or social environment. Stigma, discrimination, abuse, and attributing disabilities to supernatural causes emerged as environmental barriers to providing healthcare to CLWCPs in Ghana. All HCPs thought that the barriers in this level are the most challenging to overcome because they are mostly related to people’s perceptions, which they could not easily influence. According to HCPs, the stereotyped beliefs about disability in the community usually influence care-seeking behaviors of parents with CLWCPs. In many cases, caregivers of CLWCPs do not attempt to seek medical interventions due to a lack of awareness of biomedical explanations for disabilities like CP. Several HCPs mentioned that religious practices influence the continuity of care for CLWCPs. A common example that HCPs shared is that some caregivers often discontinue the testing and diagnosing process while others stop treatment entirely because they believe prayer is more effective and not as expensive. HCPs expressed this as a barrier to treatment, as many CLWCPs are lost to follow-up once they begin to seek care from other avenues, such as prayer camps. One HCP expressed that many parents leave medical facilities to seek a cure from religious groups because some religious leaders often present a relatable cause of the illness, unlike health professionals. This HCP said:

“*Just yesterday*, *a lady was saying that because she has a child born with Down Syndrome*… *‘Oh*, *they [CLWCPs] are seen as children who have been sent to come to do something evil*, *and so people were sending them away*. *They [family and other relatives] were sending them to the riverside*, *you know*?*’ And so*, *if people have those beliefs and if they have kids like that*, *will they come for speech therapy when they are referred*? *Will they bother to come*? *No*. *Because there is the belief that these kids are not human beings*. *Or even*, *these kids are evil*. *They have been sent to come to destroy your family*. *[When] you go to some churches*, *the pastors are saying somebody in the family was responsible for this*. *We [caregivers of CLWCPs] need to bring this amount of money to do this [religious intervention]*, *and therefore*, *they don’t even continue to come for [clinical]intervention or to benefit from the [medical] services because they are believing that it [the disability]is something someone is doing*.*” (IDI 9)*

“*There is the problem of inadequate care and abuse because the children are locked up in rooms*. *They don’t come out at all*. *They are not well-fed*. *They end up in prayer camps and some of them are made to fast*, *and all kinds of things*. *So*, *it is really a big issue*. *Even sometimes when they [caregivers of CLWCPs] come here*, *they still leave care in the hospitals and go and roam about the prayer camps and other places*, *and then come back to us when the child is much worse*. *So*, *it is a problem*, *really*: *the stigma and all that*.*”* (IDI 7)

HCPs also reported that disability-related stigma influences how health providers of CLWCPs are perceived in their community. This often discourages providers in training from specializing in the area of developmental disabilities. This was another explanation provided by HCPs for the scarcity of disability specialists in Ghana, a structural level barrier that was echoed by all HCPs. An occupational therapist noted:

“*Sometimes outside*, *you try to explain to people the kind of work you do and the kind of children you work with*, *and they try to tug you*. *And oh*! *especially*, *when are you going to find a girlfriend and your girlfriend gets to know that this [occupational therapist for CLWCPs] is the kind of work you are doing*, *it makes it look as if you are not respected*, *you do not have money and none of that*. *So that is how it is*. *That is how it is affecting [us] because people outside get to know that oh this is the kind of job you do*. *This kind of job is demeaning but I have decided to do this kind of job and it is my interest*, *I am doing it out of interest*.*”* (IDI 4)

HCPs mentioned that several patients experienced discrimination and verbal abuse while using public transportation to get to their appointments. Some HCPs mentioned how caregivers often arrive at his office with their CLWCPs looking discouraged, hopeless, and frustrated with how they (the caregiver and the CLWCP) have been treated on their way to the facility. One of the HCPs echoed the same concerns and added that these stereotypical and discriminatory behaviors are not limited to public transportation but are rampant within the community and educational, healthcare, social, and community institutions. She said:

“*If you don’t have social services support even within the healthcare delivery system*, *it is another big challenge because the mothers need support*. *Some of them are not working because of their children*. *They cannot take them to school; schools do not accept them; they cannot leave them with other neighbors; they [their neighbors] are sort of scared*, *especially of children who drool*. *Because the perception is that*, *if you come into contact with their saliva*, *you are also likely to get the same problem or to have a child with a similar problem*. *So quite a number of carers refuse to take care of children with neurodevelopmental disabilities*. *Of course*, *it takes a lot to look after them*. *It takes your emotion*, *it takes your finances*, *it takes your time*… *So that is a huge burden on the mothers*. *So*, *if they come into the hospital and they cannot find some support or something to also ease their burdens*, *then it becomes a challenge*.” (IDI 1)

Several HCPs were emotional when they discussed the common practice of killing CLWCP. Some HCPs shared a story about a CLWCP that was killed as an act of violence or due to the local belief that the child was a spirit child and had to be returned to the spirits. They mentioned that this practice prevented many parents of CLWCPs from seeking care or discontinuing treatment because of they believed that a supernatural cause was the reason they were spending all their money on the child. One HCP described this in detail:

“*There are families who are well to do*, *whose kids have been locked up with a caregiver*, *not locked in chains*, *but locked in the house*, *and they do not go out anywhere*. *And all families wherever they come from*, *whichever part of society will complain about the fact that their church is unsympathetic and does not support them*. *And our parents have talked at length about*, *I mean [how] they are isolated*. *Wherever you come from you are isolated*. *So that is a big issue*, *and I think that impacts our parents hugely …they are stressed emotionally*, *and they are stressed financially*, *some of them*. *The families who do so [kills CLWCP]*, *it is interesting*, *because until we started offering the community clinic we did not really get involved with the real nitty gritty issues*, *like for example*, *you know one of the families*, *as I was telling you earlier*, *whose child has CP*, *actually*, *this child was taken to the village and was poisoned and killed*. *Now we would not have come into touch with those families if we were not offering the community clinic*. *So*, *I am really glad that we are offering a service to the poorest of the poor because you know it really makes you know that this is a real threat*.*”* (IDI 2)

Notably, some environmental barriers associated with local perceptions of disability cut across other levels of the adapted framework. An example is the gender (superstructural) imbalance that stems from the local perception that disabilities are passed on from the maternal lineage. Another example is the educational and social isolation that stems from the local perception that disabilities like CP are communicable. Finally, disappointment and frustration with HCPs stem from local perceptions that non-medical institutions can provide a relatable explanation and cure for developmental disabilities. Some HCPs said:

“*People believe that they [the children] may have been cursed*, *the parents might have done something wrong*, *or somebody might have done something in the family*, *and the repercussions might have been passed to the child*. *So*, *parents do have hesitations when they come in [to the clinic] with these perceptions*.*” (IDI 1)*

“Because of the spiritual nature too, mothers then are forced to go along the religious pathways. Like a man of God somewhere can heal. So, they take these children to prayer camps and other places. Because when they come here [clinic] and you [health specialist] say your child has a chronic thing, it is like this man cannot cure my child, I can go to this part [traditional or religion healer] and you know, some spend their whole livelihoods there to no avail. They stop work, income levels are reduced, and mother is spending her time in a prayer camp or whatever, or with a pastor. I think it has a big impact on them.” (IDI 6)

### Relational barriers

All HCPs discussed the significance of their relationship and interactions with parents of CLWCPs. In many cases, the level of trust and communication between parents and HCPs could serve as a relational barrier to care management for CLWCPs. Some HCPs mentioned that parents of CLWCPs often bring their children to appointments with unrealistic expectations to achieve certain milestones. A common example was expecting a CLWCP who has never walked to start walking after three to four physiotherapy sessions. When such a milestone is not achieved within the expected time, many parents are said to feel betrayed, argue with the HCPs, or in some extreme cases, discontinue all forms of medical treatment in attempts to find a faster cure for their child’s condition. For instance, a female pediatrician mentioned:

“*Because it [CP] is a chronic disease*, *it takes a toll not only on the child but on the parent as well*. *So*, *some of them start off quite nicely and then after a while*, *they are lost to follow up*.*…because they sometimes expect something more dramatic and they do not see it happening*, *so they tend to lose faith and then they are lost to follow up*.*” (IDI 1)*.

### Individual barriers

HCPs mentioned that limited provider training and self-efficacy were barriers to diagnosing and treating CP. Several HCPs noted that the health outcomes of CLWCPs in Ghana could improve significantly if more health providers specialize in developmental disabilities across the country. One of the HCPs mentioned that it is very common for health professionals who are not trained in the neurodevelopmental disability to test, diagnose, and treat CLWCPs. A female pediatrician mentioned how she slowly took on this role to offer patients whatever services she could provide with her medical knowledge. She expressed:

“*We need to train staff in the area of neurodevelopment because it is an area that does not have too many specialists*. *The interest is probably not there*, *I should say*. *It’s a big challenge*. *So*, *if you do not have people who are trained to take care of children with neurodevelopmental issues*, *then you might find that you have a population of children*, *but those to take care of them are not available*, *so we need training*…*I am a general pediatrician*. *I have not gone specifically into neurodevelopmental pediatrics*. *It is not as if I have a certificate*, *a degree*, *or something in that area but I do this just because I have worked with children for a while in that capacity*. *That is how quite a number of us had to continue because we did not want to stop the clinic all together*. *So*, *even though we are not neurodevelopmental pediatricians*, *we still carry on the clinic*.*” (IDI 1)*

### Technological barriers

This level was specifically added to the social-ecological framework to capture barriers related to the lack of technological devices and medical equipment that could improve access to and quality of health services for CLWCPs. HCPs reported technology-related barriers often centered on patient referral tools, scheduling appointments, and conducting diagnostic and evaluative tests. The following quote is from a female audiologist who expressed her frustrations about the inability to provide adequate services to her patients due to a lack of diagnostic tools. She believes that HCPs have the ability to provide life-changing services to CLWCPs but are usually unable to do so due to limited technological tools required for these procedures.

“*There is a lot of pressure on the [diagnosing] equipment and the staff too*. *There are new assessments we can do*, *but the equipment is a limitation; we do not have all the things that we need*. *Most of our tools are quite outdated*. *We just make do with what we have…Audiology is both hearing and then balance but we do more of hearing*. *We hardly do any balance assessment and management because we do not have the equipment for the balance [assessment]*. *So even during the training the focus is more of the hearing [assessment]*, *if you bring the in balance [assessment]*, *then you have to talk about the test*, *but you do not have the test*. *So training is more of the hearing assessment and then the rehabilitation*.*” (IDI 8)*

Some HCPs (especially those who often refer CLWCPs to specialists) reported lack of communication tools as another technological barrier to providing healthcare to CLWCPs. Most of their offices do not have phones to call other HCPs or schedule an appointment for a patient. HCPs mentioned that they often use their phones to refer patients or schedule appointments on their behalf. One HCP gave an example of paying for a patient’s transportation fee to go schedule an appointment for diagnostic services. This was another source of frustration for HCPs, who expressed that they should not have to use their personal resources to help patients. A female speech and language therapist who has used her phone and paid for patients’ transportation on several occasions provides a recommendation on alternative ways to schedule appointments.

“*I think it’s something that the department needs to look at because I don’t see the reason why somebody should come from afar to this hospital just to book an appointment or get a date*. *There should be another way of booking appointments*. *And via phone*, *because unfortunately we are not very IT savvy—a lot of these people [CLWCPs and their caregivers] that we meet are not IT savvy to go online*, *but the phone comes in handy*. *Everybody in Ghana*, *I want to believe*, *has a phone now*.*”* (IDI 9)

## Discussion

Among a diverse group of HCPs, we explored barriers to testing, diagnosing, referral, and treatment of CP in Ghana. Barriers were identified in all six levels of an adapted version of the SDSF. The results indicate that healthcare providers experienced more barriers at the superstructural, structural, and environmental levels. The most common barriers are centered around local perceptions of disability, gender norms and ideologies, and health system policies and infrastructure. The following section highlights key findings from the study and potential solutions to address barriers related to each finding.

One of the most important findings of our study is the extent to which local perceptions of disabilities cut across multiple ecological domains. People’s socio-cultural understanding of disability influences how they engage and interact with CLWCPs, which ultimately leads to certain attitudes and behaviors that have detrimental effects on care management for CLWCPs. In Ghana, one study found that the public perceived that CP was a result of witchcraft (40%), punishment from God/gods (12%), or a curse (10%), with many who were not aware of risk factors of CP [50]. This finding is consistent with other studies demonstrating that explanations like prenatal care conditions or genetic causes are not considered [50, 59]. These perceptions align with the moral/religious model of disability, one of the oldest models of disability where disability is often regarded as a punishment from God, a test of faith, a God-given opportunity for character development, or a gift to demonstrate a special purpose of calling [60]. Although this model of disability has lost prominence, it is still prevalent in societies where religious and supernatural beliefs influence local perceptions [20, 60]. The lasting effects of these perceptions are still visible in the lives of many CLWCPs today, including infanticide.

Nevertheless, the practice of infanticide should not be understood only through the lens of the moral/religious model [60–62]. We advocate that it should also be viewed through the concept of structural violence. Structural violence, as described by Norwegian sociologist Johan Galtung, refers to harm that is experienced as a result of biased social structures that perpetuate inequalities and oppression [63]. For CLWCPs, structural violence could manifest in the form of poverty, limited access to specialized care for CP, stigma and discrimination, or the government’s decision to prioritize funding for other health conditions. A CLWCP who is born into these conditions may become a victim of infanticide if the family is overburdened by the cost of medical interventions and believes that their limited income can be put to better use. HCPs reported that many caregivers arrive at the health facility with visible dejection and stress from experiencing stigma and verbal abuse while taking public transportation. This stress is compounded by the need to travel to multiple facilities for more tests due to limited in-house diagnostic tools, exposing caregivers to more stigma and discrimination. Such long-term exposure to stigma and discrimination and inadequate services could also lead families to practice infanticide. For example, lack of support services has also been discussed as a reason why some caregivers may consider infanticide as an escape route when overburdened by the stress, financial hardship, and other structural inequities related to providing healthcare to a CLWCP [64]. For such families, the practice of infanticide may occur out of a need for survival of the family, and not merely as a result of local beliefs about disability. This view is critical because it points to the need to address the social structures, including the healthcare structures that perpetuate violence against children and women, inadequate health infrastructure, human resources, and health insurance.

Another major finding from this study is that gender imbalances significantly impact care-seeking behaviors, often leading to poor care management for CLWCPs. We found that gender norms and ideologies reinforce genealogy and norms around conception as a woman’s responsibility in their households and communities. Some HCPs conveyed that women are often blamed by their husbands and other relatives when a child presents with symptoms of a developmental disability like CP. In many cases, husbands abandon their wives, leaving them with the sole responsibility of caregiving, which creates a caregiver burden. Caregiver burden (physical, emotional, and financial strain) leads to delayed care-seeking, loss to follow-up, and hindered continuity of care for CLWCPs [6, 15, 48, 65, 66]. This imbalance in blame further reinforces how structural violence defines the experiences of CLWCPs and their caregivers. Gender norms are based on social structural arrangements, elevating one gender over another. In this setting, structural violence robs some women and CLWCPs of the resources needed to access and stay in healthcare. This amplifies the challenges specialist providers face in providing adequate care.

Another important finding from our study is that (OOP) payments and inadequate health systems infrastructure are barriers that threaten the quality of services that HCPs can render to CLWCPs. HCPs mentioned that the NHIS does not cover the cost of several medications and medical interventions for CLWCPs, which is consistent with previous research [48]. There is evidence that OOP healthcare expenditures are a barrier many individuals with disabilities worldwide face [39, 47, 48, 66]. Consistent with other studies, we found that OOP payments associated with medications and treatments often drain the financial resources of caregivers, forcing them to forego treatments and other health services [47, 66]. As prior research indicates, we also found that inadequate health system infrastructure is a barrier to providing healthcare to people living with disabilities [67]. We also found that the necessary equipment for testing and diagnosis of CP was limited in most healthcare settings. HCPs reported that they often send CLWCPs to multiple locations to conduct diagnoses and access specialized treatments [37]. Due to the fragmentation of health services in Ghana, many families, especially those living in rural communities, struggle to access the required services promptly [15, 20]. The long wait times that caregivers experience as a result of limited access to specialized services coupled with OOP payments often discourage them from seeking timely care and maintaining recommended treatment plans for CLWCPs. Care-seeking delays could cause a late diagnosis of CP, which could lead to missed treatment opportunities for reversible symptoms.

Some mechanisms of change to address barriers that partly stem from local perceptions of disability include community organizations, support groups, human rights activism, social inclusion, and equity to address stigma and discrimination. Community-based disability education efforts could increase knowledge of disability and thereby reduce stigma and discriminatory practices against CLWCPs [65]. Contact-based strategies could be pivotal in strengthening current anti-stigma education and advocacy efforts. In contact-based programs, members of the public are exposed to and learn directly from a contact with a disability or mental illness [68]. In a study assessing the impact of education and contact anti-stigma programs, *Corrigan et al*. found a significant change in factors (improvement in pity, empowerment, coercion and segregation) reflecting mental illness stigma among study participants who watched a contact videotaped [69]. Finally, services that target families impacted by structural violence could potentially reduce intentions for infanticide by helping them access resources that may alleviate the burden of caring for a CLWCP.

Addressing gender norms that encourage men to play active roles in childcare may improve the health outcomes of children [70]. Awareness efforts about CLWCPs at the community level may target men who typically play little role in childcare in Ghana. This may change local perceptions of disability and influence men’s acceptance and involvement in caring for CLWCPs. Support groups, community organizations and researchers could make a conscious effort to include men in their activities. This could provide opportunities to learn about their experiences and identify best practices and ways to support them in caring for CLWCPs. Men who excel in their roles as caregivers of CLWCPs can be recognized as community champions to encourage involvement of other men. Finally, policies around caring for CLWCPs could benefit from adopting gender equality approaches that encourage male involvement in childcare [70–73].

Expansion of the NHIS to cover health services for CLWCPs may reduce associated costs and improve the care-seeking and overall health outcomes of CLWCPs in Ghana. HCPs proposed that having a centralized location to access diagnosis and treatment services could make CP easier to manage in Ghana [57]. Interventions utilizing peer health navigation (PHN) can help expand access and accessibility of various types of support that CLWCPs may need as it has in various other fields [36]. This can be in the form of having trained individuals aid in helping caregivers navigate the healthcare system and receive social support, long-term counseling, education (identifying symptoms, barriers, treatments, etc.), and home-based management [36].

## Conclusion

This study aimed to identify the barriers to testing, diagnosing, referral, and treatment of CLWCPs by HCPs in Ghana. Using a socio-ecological model, we have examined how different levels of influence can impact health providers’ ability to provide adequate health services to CLWCPs in Ghana. Applying a socio-ecologic model offers valuable insights for a comprehensive approach to increasing the quality of health services through interventions at multiple levels. Highlighting the significant multi-level challenges HCPs face may lead to designing and implementing interventions and policies that improve the health outcomes of children with developmental disabilities like CP in Ghana and other LMICs.

### Strengths and limitations

A strength of this qualitative study is that it provides unique insights from the perspective of health providers that specialize in caring for CLWCP. This study has generated new knowledge regarding the practice of infanticide as a manifestation of structural violence impacting families with CLWCPs. Until now, explanations for this practice have mostly been linked to local beliefs and moral/religious models of disability. Another strength of this study is that we utilized a social-ecological framework to identify and provide recommendations to addressing barriers to providing healthcare to CLWCPs. This study comes with certain limitations. Due to the limited number of providers who specialize in providing healthcare to CLWCPs in Ghana, we were only able to recruit 11 HCPs for this study. In addition, two of the HCPs did not permit audio recordings, so their interview data was collected through handwritten notes. This may have reduced the quality of information collected from these two HCPs and subsequent analysis of their interview data. Despite the small sample size, the data gathered from this study provides valuable insights into understanding the challenges health providers face in providing healthcare to CLWCPs in Ghana. One limitation of the study was its exclusive focus on barriers and not facilitators. Nevertheless, we provide recommendations on strategies that may help facilitate care for CLWCPs. Future studies should identify key factors that facilitate care for CLWCP. Furthermore, HCPs were only recruited from the Greater Accra region of Ghana. Therefore, the results of this study are not representative of the entire population of health providers in Ghana. For future studies, it will be important to recruit HCPs from other regions, specifically rural areas, to examine if experiences and influential factors vary by geographic location. Finally, a key component of using a socio-ecological model is tracing the interdependence of factors at each level, which was not fully explored in this paper. However, we identified factors that permeate through multiple levels of the adapted SDSF, such as local perceptions of disabilities. Future studies could build on our work by examining how conditions at different levels of the framework interact with each other and across all six domains.

## Supporting information

S1 Checklist COREQ checklist. (PDF)

S1 Text Inclusivity statement. (DOCX)
